# Emergence of coupling-induced oscillations and broken symmetries in heterogeneously driven nonlinear reaction networks

**DOI:** 10.1038/s41598-017-01670-y

**Published:** 2017-05-09

**Authors:** Varsha Sreenivasan, Shakti N. Menon, Sitabhra Sinha

**Affiliations:** The Institute of Mathematical Sciences, CIT Campus, Taramani, Chennai 600113 India

## Abstract

Many natural systems including the brain comprise coupled elements that are stimulated non-uniformly. In this paper we show that heterogeneously driven networks of excitatory-inhibitory units exhibit a diverse range of collective phenomena, including the appearance of spontaneous oscillations upon coupling quiescent elements. On varying the coupling strength a previously unreported transition is seen wherein the symmetries of the synchronization patterns in the stimulated and unstimulated groups undergo mutual exchange. The system also exhibits coexisting chaotic and non-chaotic attractors - a result that may be of interest in connection to earlier reports of varying degrees of chaoticity in the brain.

## Introduction

Complex patterns are observed to spontaneously emerge across a wide range of spatial and temporal scales in nature^1^. Uncovering the fundamental mechanisms driving such pattern formation will contribute significantly towards understanding self-organization in non-equilibrium systems^2^. Perhaps the most influential paradigm in this context is the reaction-diffusion mechanism^3–8^, involving the interplay of self-activation and lateral inhibition mediated by diffusion^9–12^. However, not all phenomena involving activator-inhibitor interactions arise through diffusive coupling, one of the best-known counterexamples being populations in neighboring ecological habitats coupled through intra-specific competition^13, 14^. Indeed, reaction-diffusion processes can be seen as a subset of the more general class of systems involving nonlinear interactions between spatially distributed elements. Thus, uncovering the diverse range of collective phenomena associated with non-diffusively coupled systems of activator-inhibitor units can contribute towards understanding how patterns can arise in a more general setting.

Neurobiological phenomena involving synaptically coupled neuronal populations provide some of the most varied and complex instances of nonlinear interactions resulting in spatiotemporal patterns^15^. Indeed, such coordinated collective activity is seen across several spatial scales in the brain: from the network of cortical areas where brain regions comprising 10^3^–10^6^ neurons^16, 17^ interact with each other through fiber tracts^18^, to the olfactory bulb, where around 10^3^ glomerular clusters coordinate the information received from sensory neurons at the nasal epithelium^19^. Such systems can exhibit very complex collective dynamical patterns, whose origin has been previously investigated in the context of homogeneous networks of neuronal oscillators^20^. Although such a theoretical framework was shown to permit the occurrence of complex synchronization patterns that arise via spontaneous symmetry breaking, the implications of non-uniform stimulation on the global behavior of such systems are yet to be explored. An example of hetereogeneous driving of activator-inhibitor units is the case of the olfactory bulb, wherein each glomerulus, which comprises circuits of excitatory and inhibitory neurons, is activated by a specific odorant receptor type^21^, and different smells evoke responses in different combinations of glomerular clusters^22^. Processes of this nature can potentially be understood in terms of the collective dynamics of a network of excitatory-inhibitory units coupled nonlinearly with tunable strength^23–27^. As we demonstrate here, many of the complex activity patterns that could be associated with systems of this nature can be reproduced using a minimal model that eschews much of the biological complexity specific to them. Furthermore, we note that the generality of this conceptual framework allows it to be applied beyond the context of neurobiology to phenomena as diverse as ecological interactions between prey and predator populations^28^ in multiple connected habitats and interdependencies between institutions in economic systems^29, 30^.

In this paper, we investigate the collective dynamics resulting from non-uniformly driven networks of identical nodes, each comprising excitatory and inhibitory subpopulations. The heterogeneous stimulation is implemented through external inputs being applied only to a subset of the nodes. To describe the dynamics of the individual nodes, we consider the Wilson-Cowan model^31^ - a coarse-grained description of neuronal population dynamics^32^. Furthermore, we consider the simplest connection topology, viz., coupling within and between the subpopulations of all nodes^20^. One of the novel features that we observe in this system on heterogeneous stimulation is the occurrence of coupling-induced oscillations, viz., stimuli that generate only steady-state behavior in isolated nodes can drive the network into oscillatory behavior. This arises through nonlinear interactions between the nodes, and is quite distinct from the Turing-Hopf mechanism associated with diffusively coupled systems^33, 34^. It suggests that lateral connections between nodes can allow the network to recognize weak stimuli incapable of initiating activity in an isolated cluster. Increasing the strength of coupling between the nodes results in a variety of transitions in the collective dynamics of the network, the most striking of which involves a *dynamical chimera* state. This state is characterized by the co-occurrence of qualitatively distinct dynamical behaviors in elements that are otherwise identical in their nodal properties and neighborhood structure. Strengthening the coupling results in an exchange of the broken symmetry between the stimulated and unstimulated groups of nodes. In addition, we observe that the network can converge to qualitatively distinct attractors for identical system parameters, exhibiting chaotic or non-chaotic activity depending only on the initial state. This is hence possibly the simplest neuroscience-inspired model that can reproduce behavior qualitatively similar to the reported observation of multistable chaotic activity in the brain^35, 36^.

## Results

**Figure 1:**
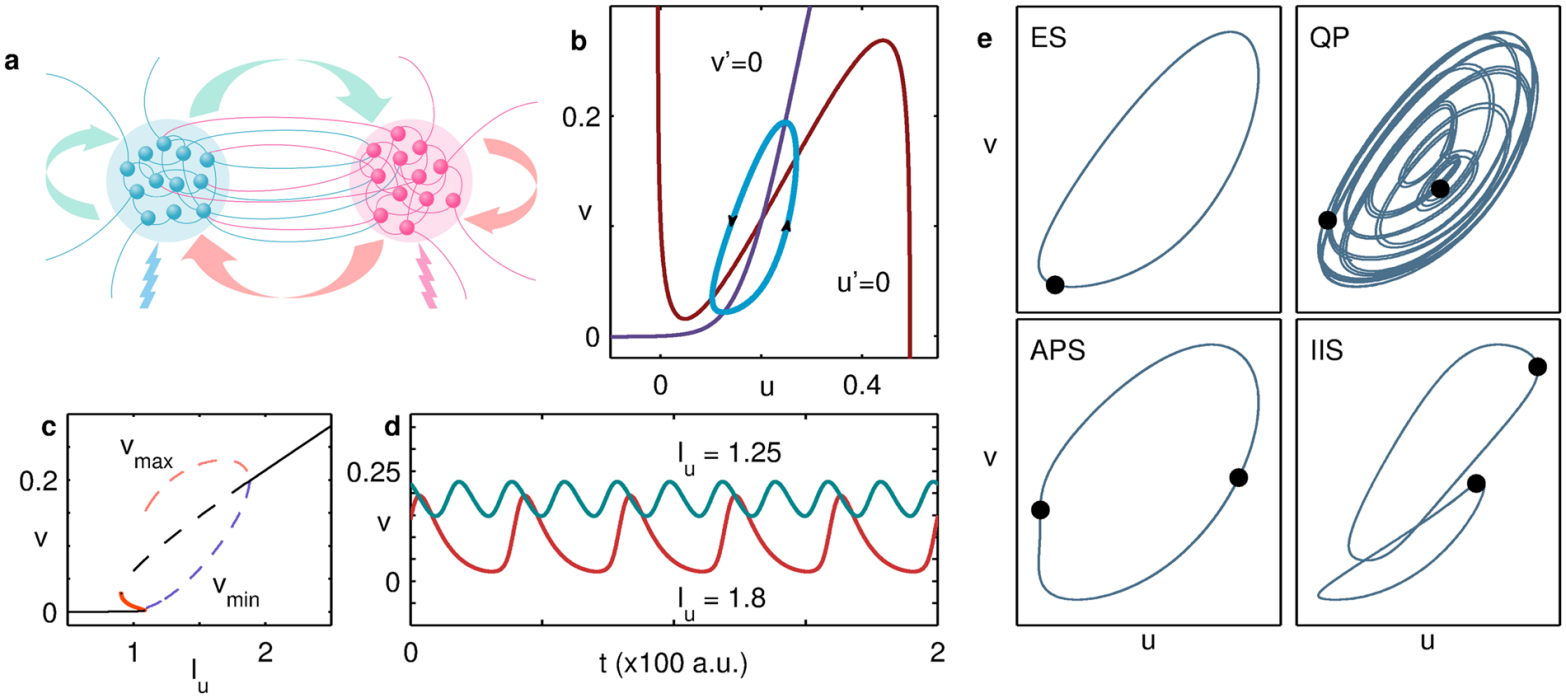
(**a**) Schematic representation of a dynamical element (node) of the network, showing the interactions between subpopulations of excitatory and inhibitory units (neurons). The lightning bolt symbols shown below each subpopulation represent the external stimuli applied to them. (**b**) Nullclines governing the dynamics of a node receiving stimulus *I*_*u*_ = 1.25 along with the resulting limit cycle attractor. (**c**) Bifurcation diagram for the inhibitory component *v* of an isolated node shown as a function of the stimulus *I*_*u*_. The broken lines indicate the unstable branch (black), as well as the peaks (*v*_max_, pink) and troughs (*v*_min_, violet) in the oscillatory regime. The solid black and thick red curves indicate the stable and saddle branches, respectively. (**d**) The *v* time-series of an isolated node receiving stimuli *I*_*u*_ = 1.25 (green) and *I*_*u*_ = 1.8 (red). (**e**) Representative phase-plane portraits for a pair of identically stimulated coupled nodes (i.e., *N*_*stim*_ = *N* = 2) in the states [L-R]: exact synchronization (ES), quasiperiodicity (QP), anti-phase synchronization (APS) and inhomogeneous in-phase synchronization (IIS). The positions of the oscillators are denoted by black filled circles. The values of the parameter set (*I*_*u*_,*w*) corresponding to the figures shown are [L-R]: (1.25, 1), (1.25, 4), (1.4, 4) and (1.8, 20).

The network that we consider is a system of globally connected nodes, each of which describes the activity of pools of excitatory and inhibitory neurons [Fig. 1(a)] (see Methods for details). On receiving a stimulus *I*_*u*_ of sufficient magnitude, a single node is capable of exhibiting limit-cycle oscillations around an unstable fixed point^31^ [Fig. 1(b)]. This limit cycle emerges via the collision of stable and saddle branches [Fig. 1(c)], and the amplitude of oscillation depends on the value of *I*_*u*_ [Fig. 1(d)]. As shown in Fig. 1(e) (for the case *N* = 2) and discussed elsewhere in detail^20^, connecting identically stimulated nodes with different coupling strengths *w* yields a rich variety of synchronization patterns including exact synchronization (ES), quasiperiodicity (QP), anti-phase synchronization (APS) and inhomogeneous in-phase synchronization (IIS) at different *w* and *I*_*u*_.

In this work, we consider heterogeneously driven networks wherein the number of nodes receiving external stimulus *N*_*stim*_ < *N*. We denote the synchronization state in such systems through the notation (*P*_*stim*_, *P*_*unstim*_), where the first and second terms correspond to the collective pattern observed in the stimulated and unstimulated nodes, respectively. For example, we denote a pattern in which the group of stimulated nodes are in IIS and the group of unstimulated ones are in ES by (IIS, ES). Note that if a group consists of only one oscillator, then by our convention it is marked as ES. Furthermore, in the pattern marked (ES, ES), while the nodes will all be exactly synchronized with members of their own group (stimulated or unstimulated), they need not be synchronized with nodes belonging to the other group.

**Figure 2:**
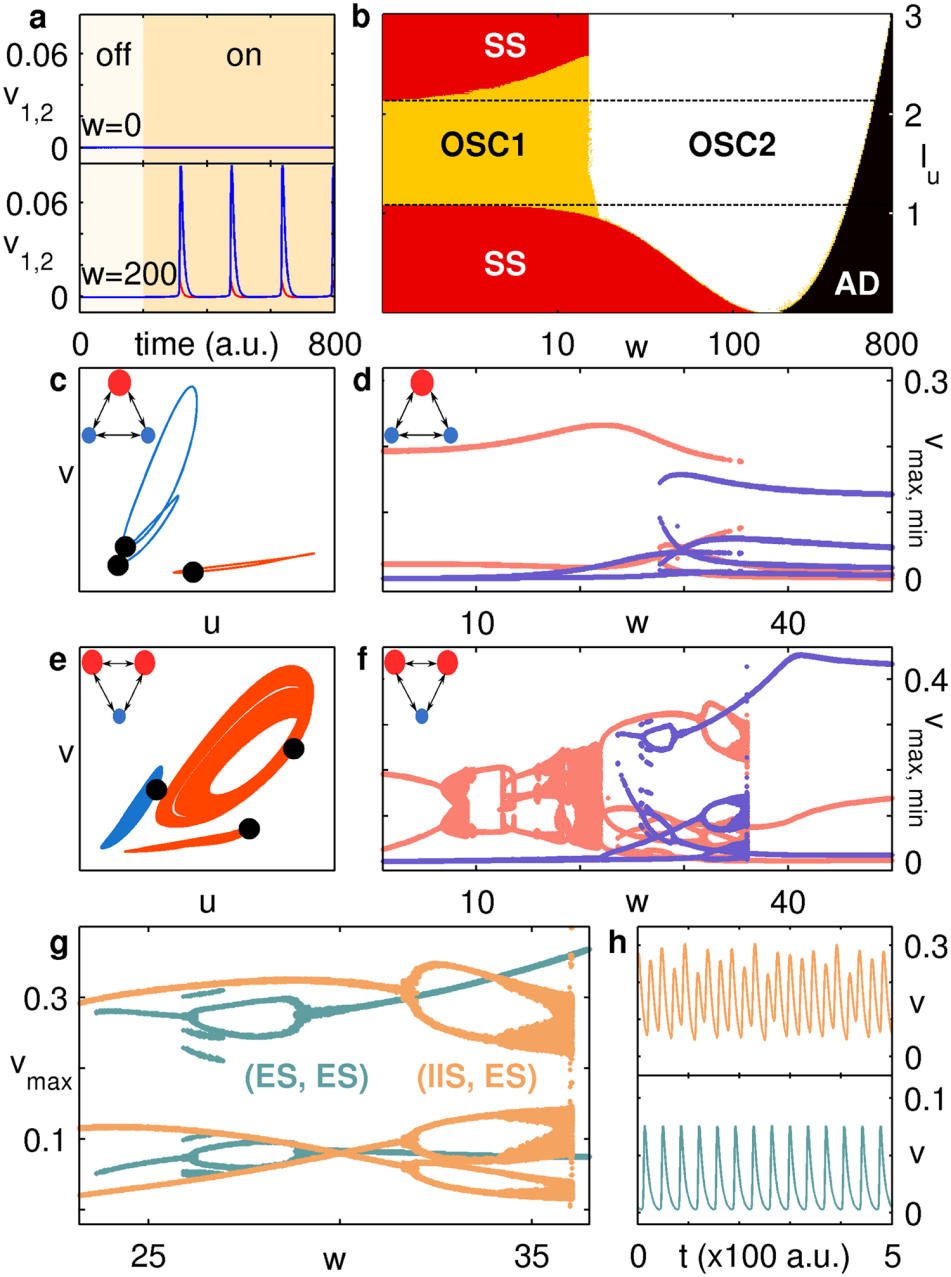
(**a,b**) Coupling-induced oscillations in a pair of nodes, where only one receives a stimulus *I*_*u*_ (i.e., *N* = 2, *N*_*stim*_ = 1). (**a**) By switching “on” a stimulus that is too weak (*I*_*u*_ = 0.1) to generate activity in the uncoupled nodes (top), oscillations can be observed by strongly coupling the stimulated (red) and unstimulated (blue) nodes (bottom). In the “off” state, the nodes receive no external input (i.e., *I*_*u*_ = 0). (**b**) The range of *I*_*u*_ for which oscillations emerge increases at higher *w*, with the region within the broken lines indicating the range of *I*_*u*_ for which oscillations are observed in an isolated node. The amplitude of the oscillations is larger for the stimulated node at low *w* (OSC1) and for the unstimulated node at high *w* (OSC2). The other dynamical regimes observed correspond to a non-zero steady state (SS) and a quiescent state characterizing amplitude death (AD). The regimes are determined via order parameters and identified as the pattern obtained from the majority (>50%) of random initial states (see Supporting Information). (**c–h**) Symmetry breaking in a system of *N* = 3 globally coupled nodes for input stimulus *I*_*u*_ = 1.25. The stimulated (large, red) and unstimulated (small, blue) nodes are indicated schematically in top-left corner of (**c–f**). Phase space projections of the trajectories (colored as per the schematic) are shown for (**c**) *N*_*stim*_ = 1, displaying (ES, IIS) state for *w* = 38 (note the broken symmetry in unstimulated nodes) and (**e**) *N*_*stim*_ = 2, exhibiting chaos for *w* = 35.6. In the corresponding bifurcation diagrams for (**d**) *N*_*stim*_ = 1 and (**f**) *N*_*stim*_ = 2, the peaks and troughs of the inhibitory component (*v*_m*ax*,*min*_) are shown as a function of *w* for the stimulated (pink) and unstimulated (violet) nodes. (**g**) Magnified view of (**f**) showing the coexistence of qualitatively distinct dynamical attractors corresponding to (IIS, ES) [orange] and (ES, ES) [green]. The system can exhibit either chaotic or non-chaotic behavior depending on its initial state, as illustrated in the top and bottom panels of (**h**) for *w* = 35.6.

The simplest case of non-uniform stimulation is when a single node receiving input *I*_*u*_ is coupled to an unstimulated node (i.e., *N* = 2, *N*_*stim*_ = 1) with strength *w*. At sufficiently large *w*, oscillations can be observed even when *I*_*u*_ lies below the range that permits limit cycle oscillations [Fig. 2(a)]. Indeed, as shown in the *I*_*u*_ − *w* parameter space diagram diagram in Fig. 2(b), at high *w* periodic activity can be observed over a much larger range of *I*_*u*_ than that capable of inducing oscillations in an isolated node. Furthermore, for lower (higher) *w* the unstimulated node has lower (higher) amplitude oscillations than the stimulated one, denoted as OSC1 (OSC2). Such *coupling-induced periodic activity* is seen even when $N\gg {N}_{stim}=1$ (see Supporting Information) and suggests that a node is capable of detecting weak, subthreshold inputs when it is coupled to one or more unstimulated nodes. Such an increase in the sensitivity to stimuli beyond the capability of a single element is an emergent collective property of the network and may be understood as an effective renormalization of the parameters governing the nodal dynamics. The observation of oscillations at larger values of *I*_*u*_, where an isolated node exhibits a stable state, may also be connected to the appearance of periodic activity in bistable systems, e.g., excitable elements subjected to a sufficiently strong stimulus, upon appropriate coupling^37^. On increasing *w* beyond a critical value that depends on the stimulus intensity *I*_*u*_, the activity of all nodes ceases, which corresponds to a state of amplitude death (AD).

A new feature, indicative of spontaneous symmetry breaking, appears on minimally increasing the size of the network to *N* = 3 keeping *N*_*stim*_ = 1. This dynamical chimera state is manifested as IIS in the unstimulated nodes [Fig. 2(c)], neither of which directly receive any external stimuli but are activated only by coupling with a common stimulated node. Nevertheless these identical nodes exhibit distinct oscillation patterns over a range of coupling strengths [Fig. 2(d)]. Note that this is different from the previously reported instance of IIS in globally coupled systems^20^, as here symmetry breaking occurs only within the group of (un)stimulated nodes. On stimulating a second node (i.e., *N* = 3, *N*_s*tim*_ = 2), another intriguing phenomenon emerges, viz., the *coexistence* of chaotic and non-chaotic attractors. The existence of chaotic behavior [Fig. 2(e)], which arises via a period doubling route [Fig. 2(f)] is confirmed by verifying the existence of positive Lyapunov exponents^38^. For example, at *w* = 35.6, we obtain a value of maximum Lyapunov exponent ${\lambda }_{max}\sim 0.016$ for appropriate initial conditions, indicating that the attractor is chaotic. Note that if we use a smaller *I*_*u*_, chaos can be seen in the even simpler stimulation configuration *N*_*stim*_ = 1, *N* = 3. For the case *N*_*stim*_ = 2, depending on initial conditions, it is possible to see either of two possible collective dynamical states corresponding to (ES, ES) or (IIS, ES), the latter being a chaotic attractor. As both the (IIS, ES) and (ES, ES) states have non-zero basin sizes [Fig. 2(g)] we can see either chaotic or non-chaotic behavior for identical system parameters over a range of *w* [Fig. 2(h)]. While the coexistence of multiple chaotic and non-chaotic attractors has been observed in other dynamical systems^39, 40^, to the best of our knowledge this is the simplest neuroscience-inspired model that can exhibit such behavior. Our results lend support to the hypothesis, based on experimental recordings from the rabbit olfactory system, that attractors with varying degrees of chaoticity can coexist in the brain^41, 42^.

**Figure 3:**
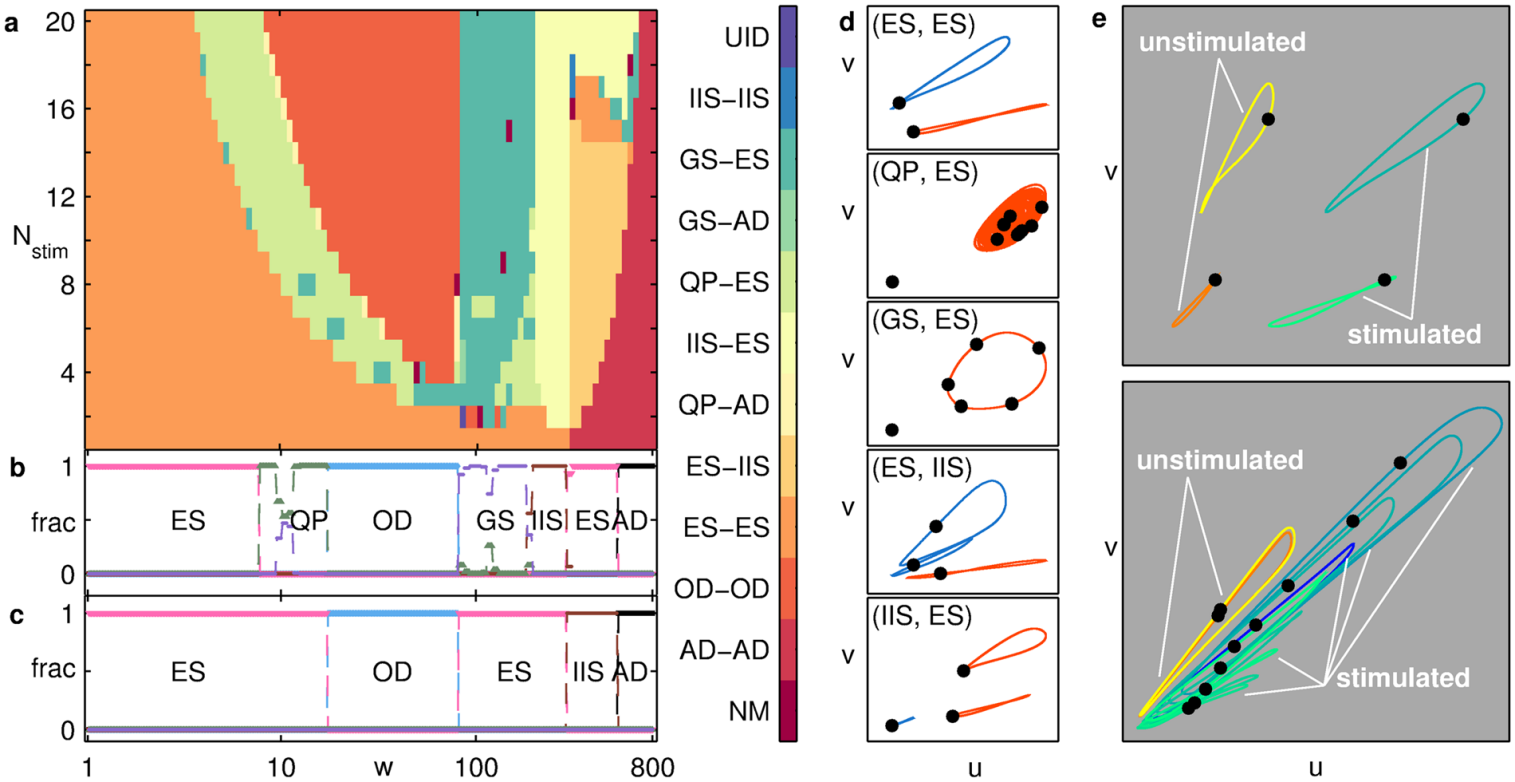
(**a–d**) Collective dynamics of a system of *N* = 20 globally coupled nodes for *I*_*u*_ = 1.25. (**a**) Different synchronization states obtained by varying the number of stimulated nodes, *N*_*stim*_, and coupling strength, *w*, with (*P*_*stim*_, *P*_*unstim*_) referring to patterns in stimulated and unstimulated groups. The colors represent the collective pattern (indicated in the colorbar) obtained for more than 50% of random initial conditions. When no pattern forms a majority, the region is indicated by NM (“no majority”), and UID (“unidentified”) indicates that the nature of the pattern was not detected by the order parameters. (**b,c**) Variation of the attraction basin size (measured as fraction of initial states reaching the attractor) with *w* at *N*_*stim*_ = 10 for the different regimes in (**a**), shown separately for the (**b**) stimulated and (**c**) unstimulated groups. Basin sizes have been estimated using 10^2^ initial conditions. (**d**) Phase space projections of the trajectories (red: stimulated, blue: unstimulated) corresponding to the different synchronization states indicated in (**a**). (**e**) Phase space projections for the pattern (IIS, IIS), corresponding to a dynamical chimera state, obtained for the case *N* = 21, *N*_*stim*_ = 18, *w* = 300, in a globally coupled system [*k* = 20, top] and in a relatively sparse network [*k* = 16, bottom]. The trajectories of the stimulated and unstimulated nodes are indicated in each case. It can be seen that as the network becomes increasingly sparse, individual nodes begin to trace distinct trajectories, thus amplifying the chimera nature of the state.

The existence of numerous synchronization regimes in the *w* − *N*_s*tim*_ parameter space becomes apparent as we increase the complexity of the system by incorporating more nodes [Fig. 3(a)]. These regimes are typically demarcated by sharp changes in the sizes of the basin of attraction of individual patterns indicated in Fig. 3(b,c). Apart from the states corresponding to ES, QP, IIS and AD described earlier, new collective dynamical patterns for the stimulated and unstimulated groups emerge. These include oscillator death (OD) which is a homogeneous non-zero steady state, and gradient synchronization (GS), a generalization of APS for systems with *N* > 2^20^. Figure 3(d) shows several of the possible collective patterns, including those corresponding to symmetry breaking (IIS) in one or both groups of stimulated and unstimulated nodes. Note that the chimera nature of the state is amplified further on making the network sparse [Fig. 3(e)], using the technique described in our earlier study of this model^20^, viz., by arranging the nodes in a circle and systematically removing links between nodes situated furthest from each other. A particularly surprising feature that we investigate in detail below is the existence of a novel transition in which the (broken) symmetry of the patterns in the stimulated and unstimulated groups undergo a mutual exchange on varying the coupling strength. This symmetry exchange manifests as a transition from the (IIS, ES) to the (ES, IIS) state [Fig. 3(a)].

**Figure 4:**
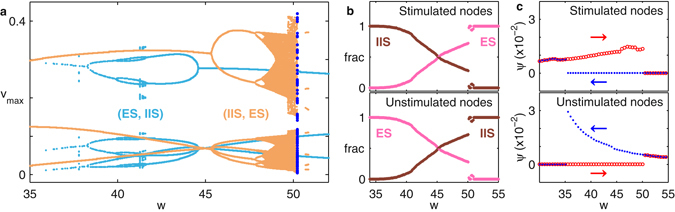
Symmetry exchange transition in a system of *N* = 4 globally coupled oscillators with *N*_*stim*_ = 2. (**a**) Bifurcation diagram showing the peaks (*v*_m*ax*_) of the inhibitory components of all nodes as *w* is varied. Distinct coexisting attractors corresponding to (IIS, ES) and (ES, IIS) are indicated by orange and light blue, respectively. (**b,c**) Variation of the (**b**) fractional basin size of these attractors and (**c**) the order parameter *ψ* = 〈*σ*_*i*_^2^(*v*_*i*_)〉 with *w*, shown separately for the (top) stimulated and (bottom) unstimulated groups. The distinct trends seen in (**c**) on increasing (red circle) or decreasing (blue dots) *w* indicate the occurrence of hysteresis-like behavior.

We examine the nature of this transition in detail by considering the simplest system in which it can be observed, i.e., for *N* = 4, *N*_s*tim*_ = 2. In this case, the states (IIS, ES) and (ES, IIS) co-exist over a range of *w* [Fig. 4(a)]. The transition between them occurs through a change in the relative basin sizes for the patterns in the two groups [Fig. 4(b)]. The mechanism of the (broken) symmetry exchange is further established by examining how the order parameter *ψ* = 〈*σ*_*i*_^2^(*v*_*i*_)〉 (non-zero values of which indicate IIS in this regime), where *σ*_*i*_^2^ is the variance across the nodes, changes upon varying *w* in either direction in an annealed manner. In this procedure, the system is allowed to evolve starting from a random initial state at low (high) *w*, following which the value of *w* is adiabatically increased (decreased). As seen in Fig. 4(c), a hysteresis-like behavior can be observed in the transition region in both groups of nodes, consistent with the mechanism of shrinking basin sizes underlying the symmetry exchange.

## Discussion

The model used here for describing the dynamics of each node has been used extensively over several decades to capture a wide range of phenomena involving neuronal populations at several length scales^32, 43^. The lack of excessive biological detail lends robustness to the model, as it facilitates the investigation of general principles underlying the appearance of complex phenomena in large neuronal networks. The model is also sufficiently general for the mechanisms reported here to potentially apply to other contexts involving interactions of activator and repressor subpopulations, such as in ecological and economic systems.

The phenomena that we report here are robust with respect to changes in both the internal coupling parameters of the model for nodal dynamics, as well as the input stimulus. The symmetry exchange transition can be observed over a large range of values of the ratio of stimuli *I*_*v*_/*I*_*u*_, and the hysteresis behavior in Fig. 4(c) can still be observed upon halving the *internal* coupling parameters within each node (see Supporting Information). The fact that symmetry-switching could be observed even at much lower internal coupling strengths points to the intriguing possibility that complex patterns observed in large networks of such oscillators are independent of the precise dynamics of the individual units. Indeed, a preliminary investigation suggests that non-trivial synchronization phenomena can be observed even when we approach the limit of no internal coupling. Further, as the number of stimulated nodes are decreased the OD state no longer appears, thus effectively resulting in a continuous transition between oscillating states on changing *w*. This suggests a further line of investigation, potentially in the context of the evolution of brain states in the transition from sleep to awareness.

Our results provide a simple framework for understanding aspects of the complex patterns of spatiotemporal activity that the brain is observed to exhibit^44^. The mesoscopic approach used here, focusing on the dynamics of a network of neuronal clusters, can yield significant insights into the mechanisms by which such patterns emerge. Furthermore, the phenomena we report here occurs in a globally connected system, which may help elucidate the important role of long-range intracortical interactions in olfactory processing^45^. Among the variety of dynamical transitions observed upon varying the coupling strength, the most striking one involves an exchange of broken and restored symmetries between the groups of stimulated and unstimulated nodes on changing the coupling strength. This result suggests an experimentally testable hypothesis, namely that stimulated and non-stimulated glomeruli may show distinct collective dynamics at different levels of arousal. This could, for instance, be realized in a set-up involving photostimulation of interconnected oscillating neural populations^46^.

To conclude, we have shown that non-uniformly driven networks of identical nodes, each comprising excitatory and inhibitory subpopulations, are capable of exhibiting surprisingly rich collective phenomena. We find that this is the simplest neuroscience-inspired system that can exhibit the coexistence of qualitatively distinct attractors (chaotic and non-chaotic) for identical system parameters. This is intriguing in light of experimental observations of chaotic dynamics of varying complexity, particularly in the olfactory system^35, 36, 41, 42, 47^, and also during transitions between interictal and ictal activity in the context of epilepsy^48^, reported over several decades and which are yet to be fully understood. Furthermore, nodes that are quiescent in isolation can spontaneously oscillate for sufficiently strong coupling, thus enabling the system to be activated even by stimuli that are incapable of generating dynamical activity in isolated nodes. These coupling-induced oscillations suggest that the spontaneous oscillations seen in networks of naturally quiescent stochastic spiking neurons^49^ can be understood as part of a larger class of phenomena characterized by the emergence of activity in quiescent systems upon coupling^50–52^. While there have been earlier reports of emergent oscillations in systems of coupled Wilson-Cowan elements^53–55^, these studies typically incorporated one or more of the following features: presence of communication delay between nodes, noise and complex connection topology obtained from structural brain data^56^. The resulting complexity of such models makes it difficult to ascertain the exact mechanism by which oscillations can arise in them. For example, it could be a consequence of the communication delay, as delay-induced oscillations are well-known even in simple systems having feedback^57^. Similarly, noise in excitable systems is known to give rise to temporal oscillations, as well as spatial patterns (such as waves) through stochastic resonance^58^. A complex connection topology introduces a further complicating factor into the investigation of the origin of such oscillatory behavior. Therefore, by choosing a system having the simplest connection topology possible (viz., a pair of coupled elements) and by not including noise and delay, we have ensured that the emergent oscillation observed in our system is exclusively coupling-induced, thereby establishing its generality and robustness. Finally, our results suggest that transitions between symmetry broken and restored symmetry states in heterogeneously driven system of coupled neural oscillators may underlie the sequence of complex activity patterns seen in the brain^44^.

## Methods

We consider a network of *N* nodes, with each node *i* comprising excitatory (*u*) and inhibitory (*v*) components that are subject to heterogeneous driving. Following the analysis in our earlier paper^20^, which was in the context of uniform stimulation, we consider interactions within, and between, all subpopulations of the nodes in our networks, as shown in the schematic in Fig. 1(a). We use one of the most appealing models for describing the behavior of interacting excitatory and inhibitory neuronal clusters proposed by Wilson and Cowan^31^ which was obtained by temporal coarse-graining of the neuronal population dynamics. The dynamical activity of each node evolves as^31^:

1 $${\tau }_{u}\frac{d{u}_{i}}{dt}=-\,{u}_{i}+({\kappa }_{u}-{r}_{u}{u}_{i}){{\mathscr{S}}}_{u}({{u}_{i}}^{in}),$$

2 $${\tau }_{v}\frac{d{v}_{i}}{dt}=-\,{v}_{i}+({\kappa }_{v}-{r}_{v}{v}_{i}){{\mathscr{S}}}_{v}({{v}_{i}}^{in}),$$

where *τ*_*μ*_ are time constants (*μ* = *u*, *v*), *u*_*i*_^*in*^ and *v*_*i*_^*in*^ are the inputs received by the respective components, ${{\mathscr{S}}}_{\mu }(x)={\kappa }_{\mu }-1+{[1+exp\{-{a}_{\mu }(x-{\theta }_{\mu })\}]}^{-1}$ is a sigmoidal response function with maximum value *κ*_*μ*_ = 1 − [1 + exp{*a*_*μ*_ *θ*_*μ*_}]^−1^, and *r*_*μ*_, *a*_*μ*_ and *θ*_*μ*_ are system parameters. As mentioned earlier, the network is globally coupled (i.e., every node has *k* = *N* − 1 links) with each link having the same weight *w*/*k*. This normalization allows our results to be system-size independent. The total inputs to each component of node *i* are *u*_*i*_^*in*^ = *c*_*uu*_ *u*_*i*_ − *c*_*uv*_ *v*_*i*_ + $\frac{w}{k}$∑_*j*_ (*u*_*j*_ − *v*_*j*_) + *I*_*ui*_ and *v*_*i*_^*in*^ = *c*_*vu*_ *u*_*i*_ − *c*_*vv*_ *v*_*i*_ + $\frac{w}{k}$∑_*j*_ (*u*_*j*_ − *v*_*j*_) + *I*_*vi*_, where *j* = 1, …, *N* (*j* ≠ *i*). To implement heterogeneous stimulation, different external inputs (*I*_*ui*_, *I*_*vi*_) are applied to different nodes. For the results shown here we have used the following set of parameter values: *c*_*uu*_ = 16, *c*_*vu*_ = 15, *c*_*uv*_ = 12, *c*_*vv*_ = 3, *a*_*u*_ = 1.3, *a*_*v*_ = 2, *θ*_*u*_ = 4, *θ*_*v*_ = 3.7, *r*_*u*,*v*_ = 1, *τ*_*u*,*v*_ = 8 and, unless specified otherwise, *I*_*v*_ = 0. We have verified that our results are robust with respect to changes in these parameter values.

## Electronic supplementary material

Supplementary Video

Supplementary Information
