# Supplementary figures and images for: Emergence of coupling-induced oscillations and broken symmetries in heterogeneously driven nonlinear reaction networks
Source: Sci Rep. 2017 May 9;7:1594. doi: 10.1038/s41598-017-01670-y (PMC5431650; doi:10.1038/s41598-017-01670-y)

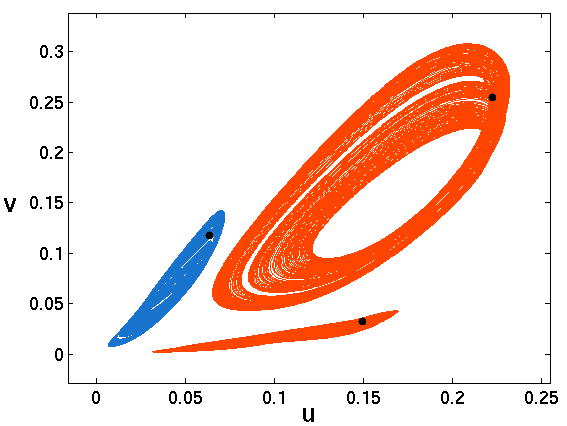

Supplement: Supplementary file 1 — Supplementary Video [file 41598_2017_1670_MOESM1_ESM.gif]
