# Supplementary material for: Emergence of coupling-induced oscillations and broken symmetries in heterogeneously driven nonlinear reaction networks
Source: Sci Rep. 2017 May 9;7:1594. doi: 10.1038/s41598-017-01670-y (PMC5431650; doi:10.1038/s41598-017-01670-y)
Supplement: Supplementary file 2 — Supplementary Information [file 41598_2017_1670_MOESM2_ESM.pdf]

SUPPORTING INFORMATION FOR

"Emergence of coupling-induced oscillations and broken symmetries in heterogeneously driven nonlinear reaction networks"

Varsha Sreenivasan, Shakti N. Menon and Sitabhra Sinha
The Institute of Mathematical Sciences, CIT Campus, Taramani, Chennai 600113, India

CONTENTS

1. Order parameters
2. Table S1
3. Figure S1
4. Robustness of Results
5. Figure S2
6. Figure S3
7. Figure S4
8. Numerical Simulations
9. Description of video `WC_chaos.gif`

ORDER PARAMETERS

The identification of each collective dynamical state is done by measuring a set of order parameters and then using the decision tree in Fig. S1 to classify the synchronization state. The same procedure is applied independently for the stimulated and the unstimulated groups of nodes. At each numbered decision point, threshold values (Table S1) for the order parameters are employed to answer the following questions:

1. Whether the magnitude of the inhibitory component of each oscillator changes over time. For a non-oscillating state, this quantity should be below a threshold ε_0.
2. Whether the magnitude of the inhibitory component of each oscillator is close to zero for all oscillators. If this quantity is below a threshold ε_1, it signifies amplitude death.
3. Whether the temporal mean of each oscillator is the same. If the difference is below a threshold ε_2, it signifies oscillator death; otherwise it is an inhomogeneous steady state.
4. Whether all the oscillators are in phase. If the difference between the phases of the oscillators is less than a threshold ε_3, it corresponds to exact synchronization.
5. Whether the temporal mean of each oscillator is the same. If the difference is greater than a threshold ε_4, it signifies inhomogeneous in-phase synchronization. In the oscillating case, once phase synchronization has been established, this order parameter checks if all oscillators have the same trajectory.
6. Whether the total amount of phase space covered by the trajectory is small. For the case of GS, this quantity is less than a threshold ε_5; if the quantity is greater than the threshold, the state is identified as QP which, by definition, completely fills a bounded region in phase space.

Threshold	Value (Stimulated)	Value (Unstimulated)
ε_0	10^{-7}	10^{-15}
ε_1	10^{-10}	10^{-10}
ε_2	10^{-10}	10^{-15}
ε_3	10^{-9}	10^{-12}
ε_4	10^{-4}	10^{-5}
ε_5	2×10^4	2×10^4

TABLE S1. Thresholds for the order parameters used to distinguish the synchronization patterns (see Fig. S1). To account for the fact that unstimulated nodes typically exhibit much smaller oscillations, the chosen threshold values are in general different for the stimulated and the unstimulated groups.

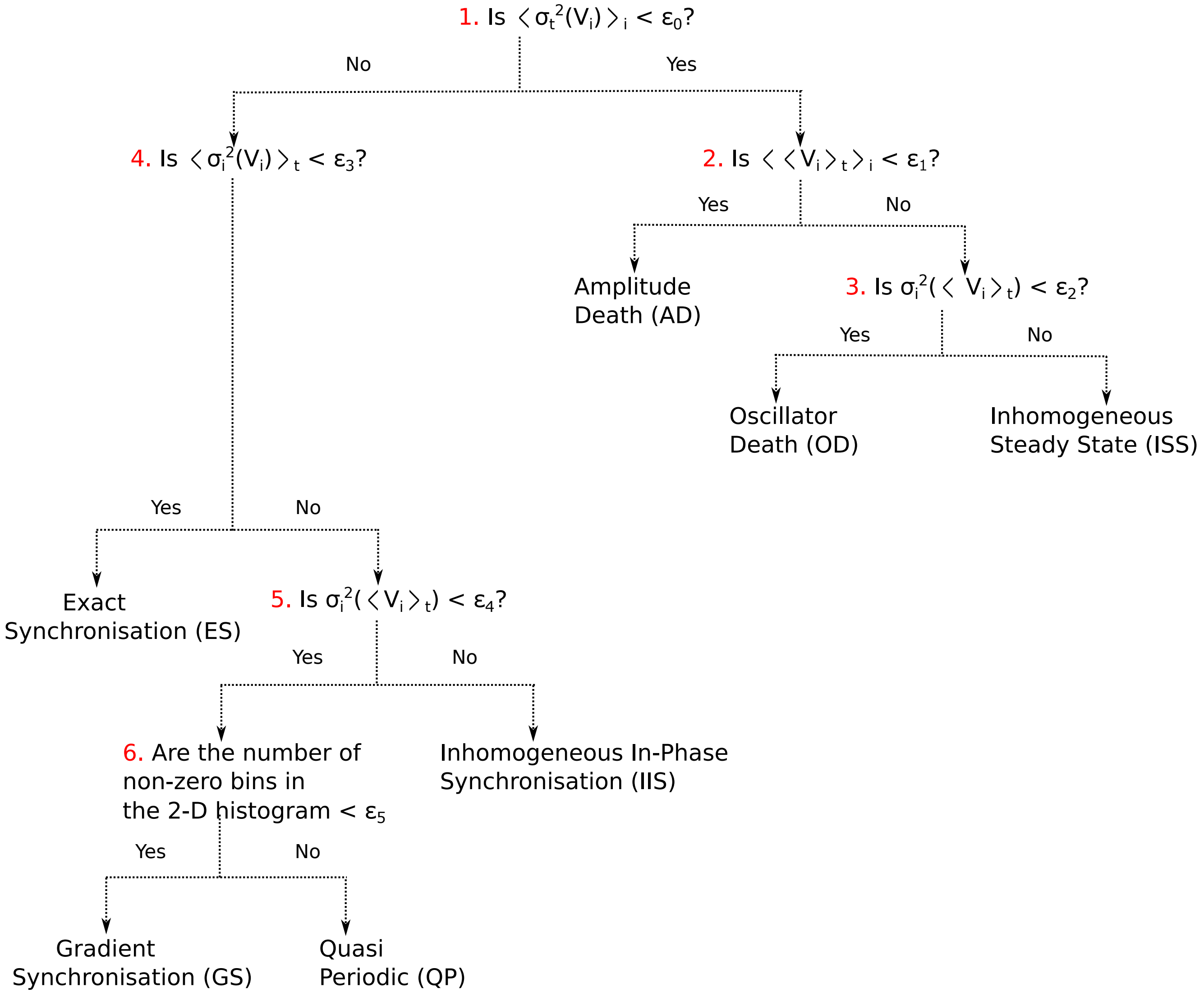

FIG. S1. Decision tree for the use of order parameters to distinguish between the different collective dynamical states. Although this tree is identical for both stimulated and unstimulated oscillators, the threshold values chosen for identifying the corresponding patterns are different (see Table S1).

ROBUSTNESS OF RESULTS

In the following, we demonstrate that the results presented in the main text are robust with respect to small changes in the system parameters or system size. First, we consider the case of coupling-induced oscillations, shown in Fig. 2 (a) of the main text. As seen in Fig. S2, steady state behavior is exhibited by a globally coupled system of N $(= 3, 5)$ nodes, with only $N_{\rm stim} = 1$ of them receiving a weak input stimulus I_u. Although the stimulation is too weak to initiate activity in an isolated node, upon coupling these nodes with sufficient strength w, we find that oscillations emerge, as in the case $N = 2$ considered in the main text.

Next, we consider the symmetry switching transition discussed in the main text. In Fig. 3 (e) of the main text, we see that for the case $N = 4$, $N_{\rm stim} = 2$ there exists a range of values within which the distinct collective dynamical states (ES, IIS) and (IIS, ES) coexist. As seen in Fig. 3 (f), the likelihood of obtaining the pattern pair (IIS, ES) is higher at lower values of coupling strength w, and only (ES, IIS) is obtained above a critical value of w. These results hold even for much larger system sizes, as shown in Fig. S3, which displays a bifurcation diagram for the case $N = 20$, $N_{\rm stim} = 10$. As we can see, the pattern pair (IIS, ES) is seen for lower w, and (ES, IIS) is seen for higher w.

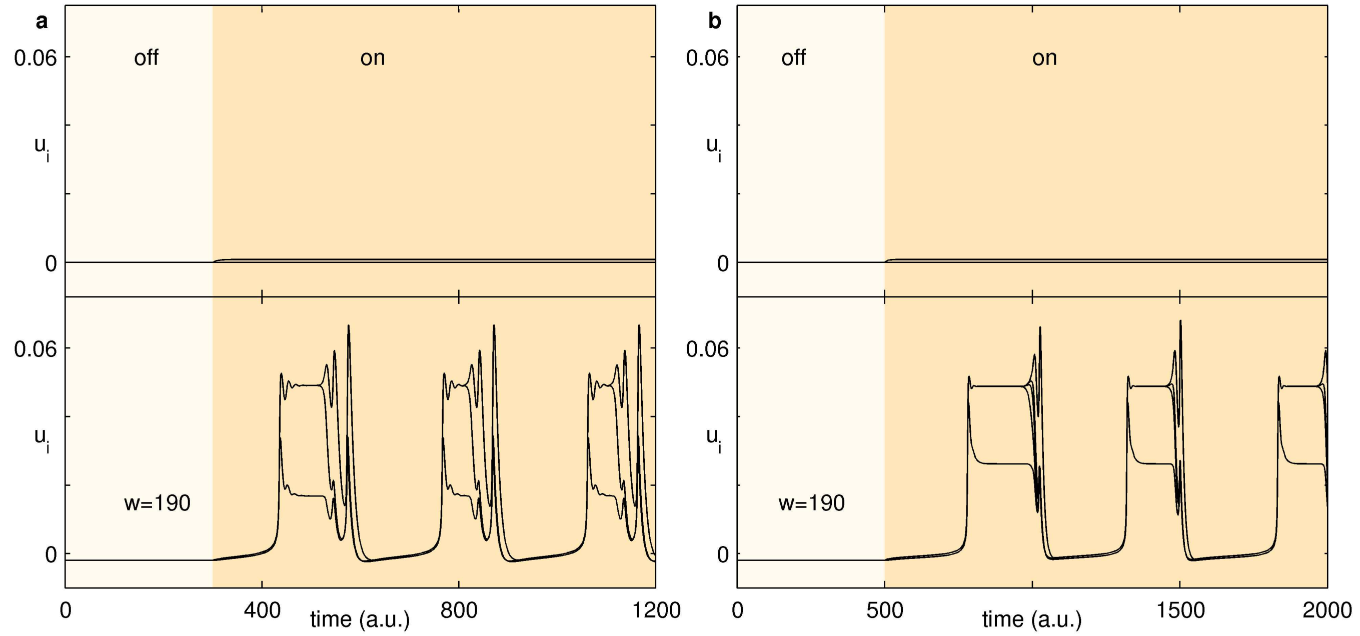

FIG. S2. Coupling-induced oscillations in a system of (a) $N = 3$ and (b) $N = 5$ nodes, where only one node receives a stimulus $I_u = 0.1$ that is too weak to generate activity in the uncoupled nodes (top). Oscillations can be observed by strongly coupling ($w = 190$) the nodes (bottom).

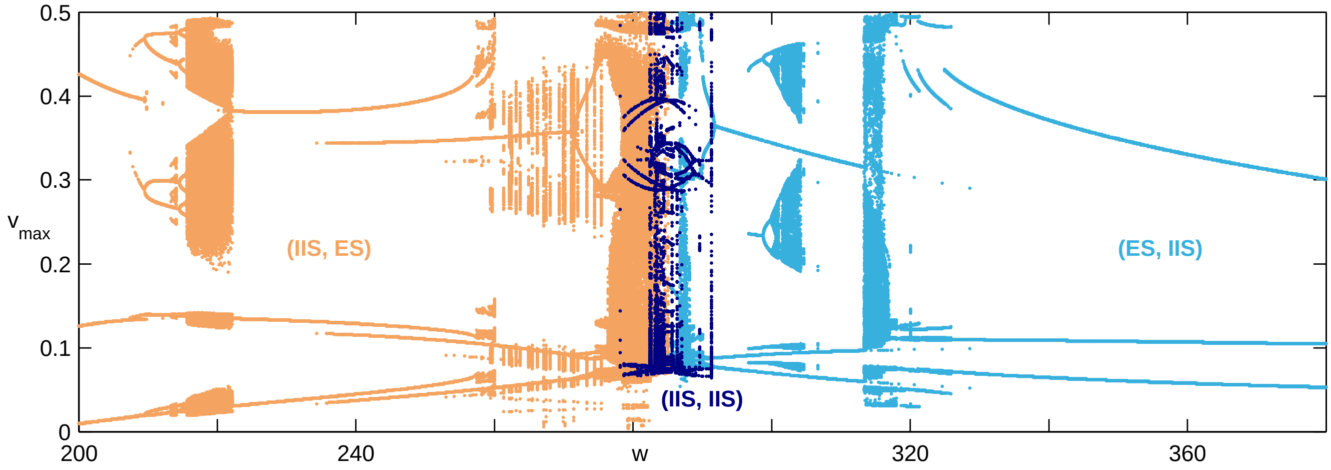

FIG. S3. Bifurcation diagram for a network with $N = 20$, $N_{\rm stim} = 10$, with the peaks ($v_{\rm max}$) of the inhibitory component, v, indicated over a range of w. Colors are used to represent different collective dynamical states, viz., orange for (IIS, ES), light blue for (ES, IIS) and dark blue for (IIS, IIS). On increasing w, a transition occurs from (IIS, ES) to (ES, IIS), corresponding to an exchange of (broken) symmetries between the stimulated and unstimulated groups.

Finally, we show that our results are robust with respect to changes in both the internal coupling parameters as well as the input stimulus applied to the inhibitory components (I_v). In Fig. S4 (a), we show a parameter space diagram indicating the synchronization states obtained upon changing w as well as the ratio I_v/I_u, for the case $N = 4$, $N_{\rm stim} = 2$. Note that, for these simulations, the values of all the internal coupling strengths $c_{\mu,\nu}$ have been halved relative to those used for the simulations reported in the main text. We find that for low values of the ratio ($\lesssim 0.25$) the patterns observed, and their phase boundaries, are almost unchanged from the case $I_v = 0$. At larger values of I_v, the phase boundaries change, but we continue to observe the symmetry switching transition from (IIS, ES) to (ES, IIS). Furthermore, as shown in Fig. S4 (b), we continue to observe a hysteresis-like behavior in the range

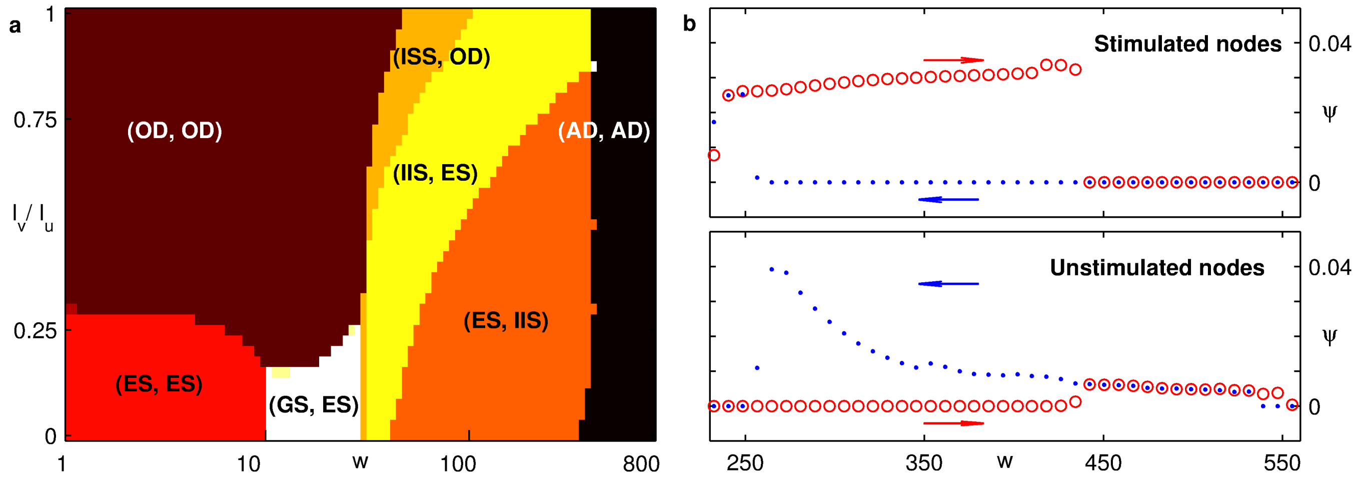

FIG. S4. Collective dynamics of the system on changing model parameters, viz., halving the internal coupling strengths $c_{\mu,\nu}$, show results qualitatively similar to those described in the main text. (a) Collective dynamics of a system of $N = 4$ globally coupled nodes, with $N_{\rm stim} = 2$, showing the different synchronization states obtained by varying the coupling strength w and the ratio of the input stimuli received by the inhibitory and excitatory components I_v/I_u (fixing $I_u = 1.25$). (b) Variation of the order parameter $\psi = \langle {\sigma_i}^2(v_i) \rangle$ with w for a network with $N = 40$, $N_{\rm stim} = 20$ ($I_u = 1.25$, $I_v = 0$). The behavior of the (top) stimulated and (bottom) unstimulated groups are shown separately. The distinct trends seen on increasing (red circle) or decreasing (blue dots) w indicate the occurrence of hysteresis-like behavior.

of coupling strengths around the symmetry-switching transition even at large system sizes ($N = 40$, $N_{\rm stim} = 20$). These observations suggest that the results reported in the main text are robust with respect to small variations in the parameter values and system size.

NUMERICAL SIMULATIONS

The equations corresponding to the system of N globally coupled nodes are solved numerically using the variable order method for stiff differential equations as implemented in the `ode15s` routine in MATLAB®. The time-series data used for analysis is recorded after allowing sufficient time for any transient effects to dissipate (typically 2×10^4 time units).

DESCRIPTION OF VIDEO

The movie `WC_chaos.gif` is generated from snapshots of the (u, v) phase space projections of $N = 3$ coupled WC oscillators with $N_{stim} = 2$ and $w = 35.6$. The colored curves represent a temporal segment of the long time behavior of each oscillator, representing their respective chaotic attractors. The initial position of each oscillator in (u, v) phase space is chosen randomly from the unperturbed limit cycle. A caption for the video is shown below:

- `WC_chaos.gif`

  Chaotic trajectories in the (u, v) phase plane for a system with $N = 3$ nodes having $N_{stim} = 2$ and $w = 35.6$. Red trajectories correspond to the stimulated nodes and blue corresponds to the unstimulated node.
